# Anticoagulation in the Elderly

**DOI:** 10.3390/ph3123543

**Published:** 2010-12-10

**Authors:** Helia Robert-Ebadi, Marc Righini

**Affiliations:** Division of Angiology and Hemostasis, Department of Internal Medicine, Geneva University Hospital and Faculty of Medicine, 4 rue Gabrielle Perret-Gentil CH-1211 Geneva 14, Switzerland; E-Mail: marc.righini@hcuge.ch (H.R-E.)

**Keywords:** anticoagulation, elderly patient, vitamin K antagonist, hemorrhagic risk, factor-Xa inhibitor, thrombin inhibitors

## Abstract

Management of anticoagulation in elderly patients represents a particularly challenging issue. Indeed, this patient population is at high thromboembolic risk, but also at high hemorrhagic risk. Assessment of the benefit-risk balance of anticoagulation is the key point when decisions are made about introducing and/or continuing such treatments in the individual elderly patient. In order to maximise the safety of anticoagulation in the elderly, some specific considerations need to be taken into account, including renal insufficiency, modified pharmacodynamics of anticoagulants, especially vitamin K antagonists, and the presence of multiple comorbidities and concomitant medications. New anticoagulants could greatly simplify and possibly increase the safety of anticoagulation in the elderly in the near future.

## 1. Introduction

The prevalence of medical conditions at risk for venous or arterial thrombosis increases gradually with age. Elderly patients are therefore more likely to require anticoagulation therapy at some point, either on a short or a long term basis. The most frequently encountered indications for anticoagulation in this category of patients are atrial fibrillation (AF), with a prevalence of approximately 10% in patients over 80 years of age [1], and the prevention and treatment of venous thromboembolism (VTE). Indeed, the incidence of deep vein thrombosis (DVT) and pulmonary embolism (PE) increases almost exponentially with age, and the majority of all VTE events occur in patients over 70 years of age [2]. In this article, we first review the different indications for anticoagulation treatments, which are basically the same as in other age categories. Then, specific considerations to bear in mind when prescribing anticoagulants in the elderly are discussed, as well as their implication for each category of anticoagulants. Finally, some future perspectives provided by new anticoagulants are presented.

## 2. General Indications for Anticoagulation

Although the prevalence of medical conditions carrying a thromboembolic risk is higher in older than in younger patients, the actual indications for anticoagulation are basically the same in all age groups and there are no data specifically focused on the elderly. Four major clinical situations warrant introduction of anticoagulant therapy: VTE prophylaxis, VTE treatment, AF and valvular heart disease. However, when stratifying the risk of thromboembolism in these different clinical settings, older age is often independently associated with a higher risk. 

### 2.1. Venous Thromboembolism (VTE) Prophylaxis

There is an overall tendency to under-use prophylactic anticoagulation in elderly medical inpatients, which seems to be more based on the physicians’ fear of higher bleeding risk than on objective data [3,4]. Among elderly medical inpatients, older age (≥75 years) is known to be an independent risk factor for VTE with an odds ratio of 1.5 for every 10 years of increase in age [5]. In a study on 852 elderly patients in subacute medical units, DVT prevalence was 15.8% with systematic lower limb ultrasound, and prevalence of *proximal* DVT was of 5.9%, in spite of a 56.1% rate of prophylactic anticoagulant therapy [6]. Assessing the need for VTE prophylaxis seems therefore even more important in older than in younger medical inpatients. Overall, the benefits of VTE prophylaxis in elderly inpatients often outweigh its risks, provided some basic precautions are observed. In surgical patients, VTE risk seems to be more related to the type of surgery than to age [7]. The latest Evidence-Based Clinical Practice Guidelines of the American College of Chest Physicians (ACCP) published in 2008 for VTE prophylaxis in hospitalized patients suggest the use of low molecular weight heparins (LMWH), unfractionated heparin (UFH) or fondaparinux for all patients apart from those considered at low risk for VTE (<10% without thromboprophylaxis), represented by cases of minor surgery in mobile patients and medical patients who are fully mobile [8]. One can easily infer that elderly patients are less likely to fall into this latter subgroup of low risk patients.

### 2.2. Venous Thromboembolism (VTE) Treatment

Unless there is an absolute contraindication, anticoagulation at therapeutic doses should be initiated as soon as the diagnosis of DVT or PE is objectively confirmed, as well as in patients with a high probability of DVT or PE while awaiting the outcome of further diagnostic tests. This initial phase of treatment consists of subcutaneous LMWH, subcutaneous fondaparinux, or intravenous/subcutaneous UFH with a grade 1A level of recommendation for all these substances in the latest Evidence-Based Clinical Practice Guidelines of the ACCP. The initial treatment is then overlapped and followed by a vitamin K antagonist (VKA) [9]. The average age of patients’ population being usually much lower in clinical trials of antithrombotic therapy in VTE than in AF, one might be reluctant to directly extrapolate the results of VTE trials to elderly patients, especially because of a fear of bleeding consequences. However, if fatal outcomes are considered, even nonagerians presenting with acute PE benefit from anticoagulation, as the incidence of fatal PE is by far greater than that of fatal bleeding complications in these patients (5.9% *versus* 2.2% in an analysis of nonagerians included in the RIETE registry) [10].

The duration of anticoagulation treatment remains a matter of debate in many situations. In cases of VTE associated with a transient and reversible risk factor such as surgery or trauma, a limited duration of anticoagulation is now widely considered to be sufficient. As anticoagulation for a period of 3 to 6 months had previously been shown to be superior to a shorter course of 4 to 6 weeks in terms of VTE recurrence rate [11,12], a limited duration of 3 months is now recommended in the ACCP guidelines in case of proximal DVT or PE associated with a major transient risk factor [9]. In cases of cancer-related VTE, in view of a high risk of recurrence, anticoagulation should be continued until the neoplasia is resolved. In these cases, LMWHs have been shown to be more effective than a VKA. Whenever possible, LMWH should therefore be continued for at least 3 to 6 months, followed either by VKA of LMWH depending mainly on the patient’s tolerance to long term subcutaneous injections [9,13]. In patients with recurrent VTE events, long-term anticoagulation is usually recommended. Indeed, a study of patients with a second episode of VTE showed a significant reduction of VTE recurrence on long-term anticoagulation as compared to 6 months of treatment, with only a non-significant trend towards increased major bleeding at 4 years of follow-up [14]. Defining the duration of anticoagulation after a VTE event without any triggering factor (also called unprovoked or idiopathic) or associated only with a minor risk factor represents a highly challenging issue. 

The latest ACCP guidelines recommend “at least 3 months” of anticoagulation in presence of an idiopathic venous thromboembolic event, followed by evaluation of the benefit-risk ratio of long term oral anticoagulation in all patients [9]. Many physicians find this recommendation difficult to apply in practice. Indeed, long term anticoagulation is known to be effective in preventing VTE recurrence, with very low event rates, 1.3% at 1 year and 2.6% at 4 years in two studies published at end of 1990s by Kearon *et al.* and Schulman *et al.* respectively [14,15]. This benefit was however obtained at the expense of an increased rate of hemorrhagic events. Although a low-intensity regimen of VKA with a target International Normalized Ratio (INR) of 1.5-1.9 proved less effective than conventional-intensity with a target INR of 2.0-3.0 (recurrence rate 1.9 per 100 patient-years *versus* 0.7 per 100 patient-years, HR 2.8) in the ELATE study [16], it still offered a significant protection against VTE recurrence compared to placebo (recurrence rate 2.6 per 100 patient-years *versus* 7.2 per 100 patient-years, with a HR 0.36) in the PREVENT study [17]. In the ELATE study, the low-intensity regimen did not offer any benefit in terms of hemorrhagic risk compared to conventional-intensity. However, the absolute bleeding rates were extremely low in both groups. In particular, the bleeding rate in the conventional-intensity arm was much lower than the rates mentioned in other studies on VKAs, and could probably not be extrapolated to every day clinical practice, especially in elderly patients. Therefore, if a given patient has an estimated high risk of VTE recurrence but also a significant hemorrhagic risk, after the initial 3 to 6 months of conventional-intensity anticoagulation with a VKA, reducing the intensity to a target INR of 1.5-1.9 could be considered on a case to case basis in order to reduce hemorrhagic risk while maintaining some protection against VTE.

### 2.3. Atrial Fibrillation (AF)

Prevention of stroke or systemic (non central nervous system) embolism in patients with atrial fibrillation represents the most frequent indication for long term anticoagulation in the elderly population because of the high prevalence of AF in this population as mentioned above [1]. AF is an independent predictor of stroke and accounts for up to 15% of ischemic strokes in the United States [18]. The absolute risk of ischemic stroke is around 4.5% per year in patients without anticoagulation. This risk can be reduced to 1.4% per year on adjusted-dose VKA [19], representing a number needed to treat of 32. However, the absolute risk of stroke varies widely between individual AF patients. Estimating stroke risk is thus a critical step in the assessment of the benefit-risk balance of chronic anticoagulation in all patients with AF. Interestingly, increasing age has been included as an independent predictor of stroke in different clinical scores developed to help the physician stratify the thromboembolic risk associated with AF. Fang *et al.* applied five of these scores to the ATRIA (AnTicoagulation and Risk Factors In Atrial Fibrillation) study cohort. They demonstrated a comparable discriminatory ability between the different schemes, albeit low for all of them (c-statistics ranging from 0.56 to 0.62) [20]. Furthermore, the proportion of patients attributed to each risk category varies greatly depending on the score that has been used [20,21].

Given the high prevalence of AF patients worldwide, identifying an optimum scheme for improving selection of high risk patients and standardizing recommendations on anticoagulation is of utmost importance. In the meantime, the widely used CHADS2 score remains a very useful tool when assessing the benefit-risk ratio of anticoagulation in everyday practice, and has been prospectively validated in a large cohort of elderly patients aged 65 to 95 years (Table 1) [22]. Lip *et al.* published a new scoring system this year under the acronym CHA2DS2-VASc, based on the Birmingham 2009 scheme, adding three new risk factors to the “classical” CHADS2 score, namely *V*ascular disease, *A*ge 65-74 years and *S*ex category [23]. Its ability to predict thromboemoblic risk was however only marginally better than CHADS2 in a cohort of patients from the Euro Heart Survey for AF included in this study. Its main interest could possibly be a better identification of patients who are truly at low risk (Table 1) [23], and its real clinical value will need to be defined with the results of further validation studies. The latest ACCP Evidence-Based Clinical Practice Guidelines for antithrombotic therapy in AF recommend oral anticoagulation with VKA in high risk patients (Grade 1A), VKA or aspirin in intermediate risk patients (Grade 1A and 1B respectively) with a preference for VKA (Grade 2A), and aspirin in low risk patients (Grade 1B). Risk categories are defined by the presence or absence of several risk factors, which are the same as those included in the CHADS2 score [24]. Overall, anticoagulation is considered to be more effective than aspirin in preventing stroke in elderly patients [25], provided there are no contra-indications to anticoagulation. Furthermore, because of the potential inconveniences and burden of long term anticoagulation, patient’s preference should be taken into account.

The general tendency to underuse anticoagulants in elderly patients is also true for AF, despite the fact that results from clinical studies are more readily applicable in real life in geriatric patients with AF, as they represent the majority of the population included in AF clinical trials, cohorts or databases. A recent retrospective analysis of more than 170,000 patients using US databases showed that less than 50% of patients with AF receive anticoagulation, with no significant difference in the rate of prescription according to CHADS2 score [26]. A systematic review of studies on current practices for stroke prevention in AF also demonstrated a consistent underuse of anticoagulants, even in high-risk patients [27]. Interestingly, in a study conducted recently in 807 frail elderly outpatients with a mean age of 81.7 years (± 7.4 years), the only item independently associated with the likelihood of not receiving VKAs in the multivariate analysis was increasing age. No other single factor, including the presence of contraindications to oral anticoagulation influenced prescription of oral anticoagulation significantly [28]. As AF is an indication for long term anticoagulation, assessing the benefit-risk ratio of anticoagulation at initiation of treatment and at regular intervals thereafter is mandatory, but represents a highly challenging issue. Specific aspects related to bleeding risk and its stratification will be discussed below in the section on vitamin K antagonists. 

**Table 1:** Stratification of thromboembolic risk in AF: CHADS2 [22] (risk of stroke) and CHA2DS2-VASc [23] (risk of stroke or other thromboembolism).

CHADS2 score*	Stroke rate per 100 patient-years (95% CI)
0	1.9 (1.2-3.0)
1	2.8 (2.0-3.8)
2	4.0 (3.1-5.1)
3	5.9 (4.6-7.3)
4	8.5 (6.3-11.1)
5	12.5 (8.2-17.5)
6	18.2 (10.5-27.4)
CHA2DS2-VASc score**	**Stroke or other thromboembolism rate per 100 patients-years (95% CI)**
0	0 (0-0)
1	0.6 (0.0-3.4)
2	1.6 (0.3-4.7)
3	3.9 (1.7-7.6)
4	1.9 (0.5-4.9)
5	3.2 (0.7-9.0)
6	3.6 (0.4-12.3)
7	8.0 (1.0-26.0)
8	11.1 (0.3-48.3)
9	100 (2.5-100)

* CHADS2 score is calculated by adding 1 point for each of the following: recent Congestive heart failure, Hypertension, Age ≥ 75 years, Diabetes mellitus; and 2 points for prior Stroke/transient ischemic attack. ** CHA2DS2-VASc score is calculated by adding 1 point for each of the following: recent Congestive heart failure, Hypertension, Diabetes mellitus, as well as Vascular disease, Age 65-74 years and Sex category (female gender); and 2 points for each of the following: prior Stroke/transient ischemic attack/thromboembolism, Age ≥ 75 years.

### 2.4. Valvular Heart Disease

Mechanical prosthetic heart valves are well known to be associated with a high risk of thromboembolism. As an example, the annual incidence of thromboemoblic events for St Jude valves is 12% and 22% for the aortic and mitral positions, respectively [29]. Anticoagulation therapy with a VKA is recommended for all mechanical heart valves in the latest ACCP guidelines, with different target INRs depending on the type and position of the valve. In patients with bioprosthetic valves in the mitral position, a limited duration of VKA therapy is recommended for the first three months after insertion, followed by long term aspirin 50-100 mg per day if the patient has no other indication for anticoagulation. For patients with rheumatic mitral valve disease, VKAs are recommended only if there is at least one additional risk factor such as AF, previous systemic embolism or left atrial thrombus [30]. For the elderly patients requiring valve replacement, a bioprosthetic valve is usually selected, since its limited durability is of minor importance and long-term anticoagulation can be avoided.

## 3. Specific Considerations in the Elderly

The different steps physicians have to go through for prescribing anticoagulants are all highly challenging when it comes to taking care of elderly patients. First, the patient’s global assessment leading to the decision that the benefits of the prescribed treatment will outweigh its risks is already a very difficult task to conduct. Then, the management of different anticoagulant molecules needs particularly careful attention in the elderly in order to avoid adverse effects. Finally, re-assessment of the benefit-risk ratio at regular intervals is of utmost importance, because elderly patients are more likely than younger patients to have additional medical conditions and medications interfering somehow with the antithrombotic regimen. Some general considerations to bear in mind while prescribing anticoagulants in geriatric patients are discussed in this section. Specific considerations for each class of anticoagulant molecules are mentioned in the corresponding sections.

### 3.1. Comorbidities and Co-Medication

Although it might seem a truism to mention that elderly patients are more likely to have comorbidities and therefore multiple prescriptions, this is a key point to consider when introducing anticoagulation in this population of patients. This is particularly true for VKAs because of these drugs’ narrow therapeutic index and multiple pharmacokinetic and pharmacodynamic potential alterations that will be further discussed hereafter.

### 3.2. Pharmacokinetics in the Elderly

With the process of ageing, body composition changes significantly with a reduction in muscle mass and total body water as well as an increase in body fat. These modifications can have an impact on pharmacokinetics of drugs as they lead to a decrease in the distribution volume of hydrophilic drugs and increase in the distribution volume of lipophilic drugs. An age-related decrease in body weight also seems to affect VKAs’ pharmacokinetics. Alterations in liver function with age are considered moderate with no significant changes in enzymatic functions in elderly patients [31]. The most significant change in organ function affecting drug pharmacokinetics is the decline in renal function [32], with an average loss in glomerular filtration rate (GFR) of 0.75 mL/min/year in healthy people with no renal disease [33]. It should be emphasized that a serum creatinine level within the normal range in an elderly patient should not falsely reassure the physician, as it can already be associated with significantly impaired renal function. In order to avoid adverse effects related to excessive accumulation of renally cleared medications, a routine estimation of renal function is recommended in all geriatric patients [34].

Two widely used equations are available: the MDRD (Modification of Diet in Renal Disease) and the Cockroft-Gault formula. Even though these formulas have not been specifically validated in large populations of geriatric patients, they provide a better estimation of renal function than the serum creatinine level, and thus are commonly used in all age groups in clinical practice. There are significant differences in renal function estimation results between these two formulas in the elderly, with an overestimation of GFR by the MDRD equation. Cockroft-Gault formula should therefore be used for detecting significant renal impairment and adjusting drug dosage if necessary. Moreover, creatinine clearance using this formula matches drug manufacturers’ dosage tables [35]. Reduced clearance of some medications by the ageing kidney prolongs their half-life, potentially leading to accumulation and toxicity if the drug is administered repeatedly. In order to avoid these adverse effects, two adjustment options are applicable: reduction of each dose or increase in the time interval between doses. These adjustment strategies, although not strongly evidence-based, can be considered for low molecular weight heparins at therapeutic dose in VTE treatment in patients with impaired renal function, by monitoring of anti-Xa levels [9].

Other factors that have an impact on pharmacokinetics are not age-specific. Diminished absorption of orally administered medications in some situations (in particular VKAs), genetic polymorphisms influencing hepatic metabolism, and drug interactions at the cytochrome P450 CYP2C9 levels are not influenced by patients’ age, but remain of high clinical relevance in the elderly as in younger patients [34,36]. Of these, drug interactions represent a major issue in anticoagulation of elderly patients with VKAs. Indeed, apart from the problem of frequent polymedication [37], elderly patients are more prone to multiple changes of concomitant drugs related to intercurrent acute illnesses, with the risk of fluctuating anticoagulation intensity (outside either end of therapeutic range) and potential adverse thromboembolic or hemorrhagic consequences.

### 3.3. Pharmacodynamics in the Elderly

Significant pharmacodynamic changes are also observed in elderly patients, represented in general by a higher sensitivity to medications [31], the mechanisms of which are not always fully understood. In the case of anticoagulants, several factors could have an impact on pharmacodynamics in the elderly. For example, unfractionated heparins bind to numerous plasma proteins and cellular components in addition to antithrombin. Variability of these determinants could contribute to the unpredictable pharmacokinetic and pharmacodynamics properties of heparins [38].

Pharmacodynamic alterations with age are most prominent with vitamin K antagonists. One of the major factors contributing to variability of response and greater sensitivity to VKAs in the elderly is poor dietary vitamin K intake, leading to a reduced competitive antagonism to the effect of VKAs. This is particularly true in acute medical settings where patients’ nutritional intake is even lower. On the contrary, over-the-counter multivitamin tablets can contain vitamin K and significantly reduce response to VKAs. Other mechanisms involved in increased sensitivity to VKAs are decreased production of vitamin K by intestinal flora in presence of broad-spectrum antibiotics, or increased catabolism of vitamin K-dependant clotting factors in hypermetabolic states such as fever. Finally, another type of pharmacodynamic interaction is concomitant intake of a medication interfering with platelet aggregation, such as aspirin or non-steroidal inflammatory drugs, frequently prescribed in elderly patients, increasing bleeding risk [39]. 

### 3.4. Risk of Falls

One of the great concerns of physicians taking care of geriatric patients is the risk for falls. In a study by Gage *et al.* [40], the incidence of intracranial hemorrhage was shown to be higher in elderly patients with AF “at high risk for falls” compared to other patients (2.8 *versus* 1.1 per 100 patient-years). Warfarin did not affect the incidence rate of this complication, but was associated with more severe events and higher 30-day mortality. However, elderly patients with AF “at high risk for falls” were at even higher risk for ischemic stroke (13.7 per 100 patient-years). Therefore, if the net clinical benefit is considered, patients with AF associated with additional stroke risk factors seem to benefit from anticoagulation even if they are at high risk for falls [40]. 

### 3.5. Hemorrhagic Risk

Bleeding and especially intracranial hemorrhage is the most dreaded complication of anticoagulant therapy. Regardless of the category of anticoagulant, increasing age represents an independent risk factor for bleeding with anticoagulation in the therapeutic range [41]. However, the individual patient’s characteristics and comorbidities can contribute to increase hemorrhagic risk. Some of these characteristics have been integrated in the different bleeding scores. Two examples of bleeding scores are presented in Table 2 [42,43]. Bleeding scores, especially those specifically developed for AF patients, are discussed in more detail in the section on VKAs. Another intuitive point, which has been largely demonstrated only for warfarin, is the increase in rates of hemorrhagic complications associated with supra-therapeutic anticoagulation. As already mentioned above, the challenge in elderly patients lies not only in the difficulty of assessing the benefit-risk ratio of anticoagulation, but also in the management of such treatments in a way that avoids undertreatment as well as overtreatment. Observing specific considerations can help to maximize the security of these treatments while maintaining efficacy. Moreover, patient information and education should be part of anticoagulant therapy as it is the case for diabetic patients. In fact, poor patient education has been proven to be a major risk factor for anticoagulation-related bleeding complications in the elderly [44]. This is particularly important with VKAs because of the numerous potential pharmacological influences of nutrition and changes in associated medications.

## 4. Different Well Established Anticoagulant Options

In this section, different established options for prophylactic and therapeutic anticoagulation will be discussed with an emphasis on specific considerations in elderly patients. These medications have all proven their efficacy in reducing the rate of thromboembolic events. All of them however have many drawbacks and are far from meeting the criteria that an “ideal” anticoagulant should meet on top of its efficacy against thromboembolism: oral administration, predictable dose-response and kinetics, low nonspecific binding to plasma proteins, no necessity for routine monitoring, wide therapeutic index, little interaction with food or other medications, low rate of hemorrhagic complications, and finally simple reversibility in case of overdose and/or bleeding [45]. Some of these criteria are fulfilled by new anticoagulant agents, which are discussed in the next section.

### 4.1. Unfractionated Heparin (UFH)

UFH consists of a heterogenous mixture of glycosaminoglycans, derived from porcine intestinal mucosa, with a mean molecular weight of 15,000 Daltons, ranging from 3,000 to 30,000 Daltons. Heparin has a unique pentasaccharide sequence that binds to antithrombin and leads to a conformational change in the latter. The heparin-antithrombin complex inactivates activated factor X (factor Xa), a key enzyme positioned at the start of the common pathway of coagulation cascade. In addition to antithrombin, heparin simultaneously binds to thrombin with an inhibitory effect on this enzyme. Heparin exerts an equivalent anti-Xa and anti-IIa activity with a ratio close to 1. Because of its poor intestinal absorption, UFH can only be administered by intravenous (iv) or subcutaneous (sc) route. It circulates in blood bound to many plasma proteins which can contribute to its unpredictable pharmacokinetics [46]. It also binds to endothelial cells and platelet factor 4, with one of its potentially severe complications being heparin-induced thrombocytopenia, an antibody-mediated adverse reaction to heparin-platelet factor 4 complexes associated with a high risk of thrombosis [47].

Although it has been largely replaced by low molecular weight heparins (LMWHs) in most clinical situations, it still has specific indications such as during intravascular catheterization procedures or in cardiovascular surgery, but also in VTE prophylaxis and treatment. It is indeed mainly in cases of severe renal failure with contra-indication to LMWHs that UFH is prescribed in these indications. The efficacy of UFH 5,000 units three times daily is comparable to that of enoxaparin 40 mg once daily in VTE prophylaxis in medical inpatients, as demonstrated in a multicentre randomized controlled study including a majority of patients over 70 years of age [48]. For VTE treatment, UFH can be prescribed intravenously or subcutaneously with activated Partial Thromboplastin Time (aPTT) monitoring. Weight-adjusted subcutaneous dosing without monitoring is also acceptable [9] if there is no other choice. Another characteristic that renders UFH preferable to LMWH in specific clinical situations is its very short half-life when administered in the iv form, allowing for rapid reversal of anticoagulant effect after stopping the infusion.

**Table 2:** Risk of major bleeding on anticoagulation: the RIETE registry bleeding score [42] and the HEMORR2HAGES score [43].

RIETE bleeding score *	Major bleeding per 100 patients within 3 months of anticoagulant therapy (95% CI)
0	0.3 (0.1-0.6)
1-4	2.6 (2.3-2.9)
>4	7.3 (5.6-9.3)
HEMORR2HAGES score **	**Major bleeding per 100 person-years (95% CI)**
0	1.9 (0.6-4.4)
1	2.5 (1.3-4.3)
2	5.3 (3.4-8.1)
3	8.4 (4.9-13.6)
4	10.4 (5.1-18.9)
≥5	12.3 (5.8-23.1)
Any score	4.9 (3.9-6.3)

* RIETE bleeding score is calculated by adding 2 points for recent major bleeding; 1.5 points for each of the following risk factors: creatinine level > 12 mg/dL (110 µmol/L) or anemia (Hb < 13 g/dL in men or 12 g/dL in women); 1 point for each of the following risk factors: cancer, clinically overt PE, age > 75 years). ** HEMORR2HAGES score is calculated by adding 1 point for each of the following: Hepatic or renal disease, Ethanol abuse, Malignancy, Older age (>75 years), Reduced platelet count or function, Rebleeding risk (= previous bleeding; 2 points), Hypertension (uncontrolled), Anemia, Genetic factors, Excessive fall risk, and Stroke/TIA.

When UFH is prescribed for initial treatment in VTE, an increased risk of bleeding and major bleeding is observed in patients ≥72 years compared with younger patients, with incidence rates of 14.1% *versus* 7.1% for bleeding and 11.1% *versus* 3.1% for major bleeding [49]. Interestingly, elderly patients have higher heparin levels and a tendency for higher aPTT with standard heparin doses not adjusted to weight, and require lower doses of heparin to achieve therapeutic aPTT levels [49]. To avoid overanticaogulation with UFH, a weight-adjusted dosing pattern should be used. In the initial treatment for VTE, an iv bolus of 80 units/kg followed by an infusion at 18 units/kg/h is recommended, with further adjustment of dosage according to aPTT level [9].

### 4.2. Low Molecular Weight Heparins (LMWH)

Low molecular weight heparins are glycosaminoglycans with approximately one third the molecular weight of UFH. They are derived from various processes of depolymerization of UFH. Different industrial preparations thus contain variable proportions of shorter and longer chains. The higher the proportion of short chains, the lesser the ability to bind to thrombin, so that the relative anti-IIa to anti-Xa activity varies from 1:1.5 to 1:3 between different LMWHs. LMWHs are administered subcutaneously and have less nonspecific binding to plasma proteins. Their bioavailability is greater and their pharmacokinetics more predictable than UFH so that monitoring is not needed. They have a half-life of 4-5 hours and their elimination is mainly renal [38]. 

LMWHs are widely used in VTE prophylaxis in medical and surgical settings. The MEDENOX trial showed superiority of enoxaparin 40 mg compared to placebo for preventing symptomatic VTE and venographically diagnosed DVT from 14.9% to 5.5% in acutely ill medical patients, representing a number needed to treat (NNT) of 11, with no increase in adverse outcomes [50]. In the same study, enoxaparin 20 mg per day did not show any benefit over placebo. Of note, around 50% of MEDENOX study population was >75 years old. A subgroup analysis of these patients demonstrated an even greater benefit from enoxaparin 40 mg with a NNT of 7 (VTE risk reduced from 18.5% to 4.1%) [51]. The PREVENT study also showed superiority of another LMWH dalteparin 5,000IU o.d. over placebo in medical inpatients, reducing incidence of symptomatic VTE and asymptomatic *proximal* DVT from 4.96% to 2.77% (NNT = 45). As for MEDENOX, a subgroup analysis of patients >75 years was also performed in PREVENT, confirming the efficacy of dalteparin in this category of patients with a lower NNT of 26 (VTE events as defined above of 4.2% *versus* 8.0%) and no significant increase in the risk of major bleeding (1.1% *versus* 0.7%) [52]. Thresholds of serum creatinine levels rather than calculated creatinine clearance (CrCl) levels were used in the exclusion criteria and were defined at a creatinine level of more than 1.7 mg/dL (150 µmol/L) in MEDENOX and more than 2 mg/dL (176.8 µmol/L) in PREVENT. In the initial phase of VTE treatment, a Cochrane database systematic review showed a global superiority of weight-adjusted fixed-dose sc LMWH compared to adjusted dose iv UFH: LMWHs were associated with a significantly lower rate of thrombotic complications (3.6% *versus* 5.4%), major bleeding (1.2% *versus* 2.0%) and death (4.5% *versus* 6.0%) [53]. 

The main concern when prescribing LMWHs in elderly patients is renal impairment. At therapeutic dosage, even mild decreases in renal function can lead to accumulation of LMWHs. When administered to healthy volunteers, once daily sc nadroparin 180 IU/kg for 6-10 days is associated with an accumulation of anti-factor Xa activity in the elderly group (65 ± 3 years; mean CrCl 62 ± 2 mL/min) but not in the younger group (25 ± 4 years; mean CrCl 114 ± 15 mL/min) in spite of similar body weights [54]. Particular attention is therefore needed in the elderly and CrCl using the Cockroft-Gault formula should be calculated in these patients before prescribing LMWHs. 

Evidence is insufficient in the literature to define an optimal creatinine clearance cut-off under which LMWHs should not be prescribed [55]. In a meta-analysis published by Lim *et al.*, analysis of 12 studies with a total of 4971 patients showed increased risk of major bleeding in patients with severe renal insufficiency receiving LMWHs with a rate of 5% *versus* 2.4% for creatinine clearance < 30 mL/min compared to >30 mL/min; OR = 2.25 (95% CI 1.19-4.27; p = 0.013) [56]. Enoxaparin was the most represented molecule in this meta-analysis. In four studies of enoxaparin used at standard dose, peak anti-Xa levels were significantly higher in patients with CrCl < 30 mL/min. This was not the case in three studies in which enoxaparin dose was empirically adjusted. Major bleeding was also increased in patients with CrCl < 30 mL/min compared to those with CrCl >3 0 mL/min at standard therapeutic doses of enoxaparin (8.3% *versus* 2.4%) but not when the dose was empirically adjusted (0.9% *versus* 1.9%; p = 0.23) [56]. Additional data will however be needed in order to confirm the efficacy and safety of adjusting enoxaparin and other LMWHs’ doses in patients with significant renal impairment.

At prophylactic doses of LMWH, renal impairment is less of a concern. Indeed, anti-factor Xa activity has been shown to be higher at prophylactic doses of enoxaparin (40 mg sc once daily) in acutely ill elderly medical inpatients with a mean age of 87.5 years, with a significant effect of creatinine clearance <30 mL/min and body weight <50 kg. However, among patients with serious hemorrhagic complications in this study (only 5 patients), anti-factor Xa levels were not higher than in patients without bleeding [57]. Because of the low number of events, the results of this study cannot conduct to any formal recommendation in clinical practice. Tinzaparin at the prophylactic dose of 4,500 IU/day seemed to show less accumulation than enoxaparin 40mg /day in elderly patients with CrCl 20-50 mL/min in a pharmacokinetic study, but the clinical relevance of this observation in not clear [58]. Based on common sense more than on evidence, it seems reasonable in elderly patients with severe renal dysfunction to prefer UFH for VTE prophylaxis or to measure anti-factor Xa level after a few doses of prophylactic LMWH, not with the aim of dose adjustment but in order to detect a tendency to accumulation. 

In general, unless there is an absolute contra-indication to LMWHs, these should be preferred to UFH in VTE prophylaxis and treatment, because of their predictable pharmacokinetics, overall greater efficacy and security (provided some precautions are observed), and lower incidence of heparin-induced thrombocytopenia. A suggested regimen for use of parenteral anticoagulants in patients with renal insufficiency is presented in Table 3. We would like to emphasize that dose reduction suggestions in this table are not based on strong evidence, and should only be applied on a case to case basis.

**Table 3:** .Suggested regimen for the prescription of heparins in elderly patients with renal failure ^#^.

Creatinine clearance (CrCl)	Prophylactic anticoagulation	Therapeutic anticoagulation
>50 mL/min	FondaparinuxLMWH LMWH	FondaparinuxLMWH LMWH
30-50 mL/min	Fondaparinux without dose reduction LMWH without dose reduction	Fondaparinux for a limited duration of treatment (caution in case of prolonged treatment if CrCl is at the lower limit of the range because of the risk of accumulation) LMWH without dose reduction, anti-Xa level after 3d or 4^th^ dose, anti-Xa monitoring +/− dose reduction if tendency to accumulation. N.B. dose reduction can be considered if CrCl is at the lower limit UFH
<30 mL/min	UFH LMWH with dose reduction (1/2 dose), anti-Xa level if prolonged treatment (to make sure there is no accumulation)* Fondaparinux contra-indicated	UFH LMWH with dose reduction (1/2 dose), anti-Xa level after the 2nd dose, and minimum twice weekly thereafter* Fondaparinux contra-indicated Fondaparinux contra-indicated

^#^ The regimens proposed here do not represent formal recommendations or guidelines, but only suggestions that could be helpful in the practical management of heparins in the elderly in everyday practice; * Both attitudes not formally validated.

### 4.3. Synthetic Pentasaccharides (Fondaparinux, Idraparinux)

Pentasaccharides are the first generation of selective anti-Xa inhibitors. Their structure consists of the pentasaccharide region of heparin molecule that binds specifically to antithrombin. They thus exert an indirect and selective inhibition of factor Xa. As they lack the longer saccharide chains that bind to thrombin, they have no direct inhibitory effect on thrombin. Several advantages over heparins need to be mentioned. Indeed, pentasaccharides are produced synthetically and not derived from animal tissues thereby conferring higher security in terms of potential contamination and allergic reactions. A second and clinically important advantage over heparins is the absence of reaction with platelet function and heparin-PF4 antibodies, with no convincing case of fondaparinux-induced thrombocytopenia described to date. Finally, they have an almost 100% bioavailability and predictable pharmacokinetics and are administered subcutaneously. Fondaparinux reaches its peak in 2 hours and has an elimination half-life of 17 h, with purely renal clearance [59], which represents an obstacle to its wide prescription in elderly patients as it is already the case with LMWHs. Moreover, no specific antidote exists, and in case of major bleeding, most authors recommend the use of recombinant factor VIIa, which was shown to reverse the anticoagulant effect in healthy volunteers [60]. 

Fondaparinux at a daily sc dose of 2.5 mg is effective and safe in preventing VTE in medical inpatients >60 years old, with an incidence of VTE (composite endpoint of DVT diagnosed by routine venography and symptomatic VTE) of 5.6% *versus* 10.5% for placebo (NNT = 20) [61]. Fondaparinux has also proven its efficacy in major orthopedic surgery [62,63]. Although it is thought to be even more effective than enoxaparin in reducing VTE, it may be associated with a slightly higher incidence of major bleeds, mainly at surgical site [64], contributing to surgeons’ reluctance to prescribe it as a first choice. Based on data from phase II trials and pharmacokinetic simulation models, Turpie *et al.* suggested that a fondaparinux dose of 1.5 mg in patients with moderate renal failure (defined as CrCl 20-50 mL/min) yielded a drug exposure comparable to that of 2.5 mg in patients with CrCl > 50 mL/min, and could improve safety of this drug while maintaining efficacy. Further investigation seems however necessary before this lower dose is recommended widely in practice [65]. In the meantime, fondaparinux should be prescribed for medical or surgical prophylaxis at 2.5 mg per day, and is contraindicated in patients with severe renal insufficiency (defined by the manufacturer as CrCl < 20 mL/min for prophylactic dosage). 

For the initial treatment of VTE, the two MATISSE trials confirmed equivalent efficacy and safety profile of fondaparinux compared with a double-blind design to twice daily 1 mg/kg sc enoxaparin in the DVT trial [66], and with an open-label design to continuous iv UFH in the PE trial [67]. Fondaparinux dose was 7.5 mg once daily for patients 50-100 kg, 5 mg for patients weighing <50 kg and 10 mg for those >100 kg. Mean age of patients in these two studies was between 61 and 63 years ± 16, and patients with a serum creatinine level above 177 µmol/L (2.0 mg/dL) were excluded. The results of these trials may therefore not be directly applicable to the very old, without some specific precautions. The manufacturer sets the limit of CrCl to 30 mL/min for the therapeutic dose, under which fondaparinux is contra-indicated. In our opinion, caution should be observed in frail elderly patients with borderline renal function with both prophylactic and therapeutic doses. Indeed, fondaparinux half-life could be increased in elderly patients [32], leading to potential accumulation in case of prolonged treatment.

Idraparinux is another synthetic pentasaccharide that indirectly inhibits factor Xa. Its half-life is considerably longer than fondaparinux requiring weekly subcutaneous injections. Two randomized open-label non-inferiority trials compared the efficacy and safety of 3-6 months idraparinux to standard therapy (UFH or LMWH followed by VKA) in patients with DVT and PE [68]. The results satisfied the prespecified non-inferiority requirement in the DVT study, but not in the PE study. This might be due to the fact that early treatment with a long half-life drug and no charging dose could not cover the higher early recurrence risk after PE. In the AMADEUS trial on AF patients, idraparinux was associated with an increased risk of major bleeding, especially intracranial bleeding compared to VKA [69]. To solve the problem of management of acute hemorrhagic complication in a drug with very long half-life, a biotinylated variant of the drug, idrabiotaparinux was developed that can be effectively neutralized by avidin [70]. Nevertheless, a long half-life molecule eliminated purely by renal route does not seem the most attractive option in elderly patients, as the antidote may not be widely available in all emergency centres.

### 4.4. Vitamin K Antagonists (VKA)

Warfarin is the most widely prescribed VKA worldwide. It is a drug from the coumarin family, achieving its anticoagulant effect by interfering with vitamin K metabolism, thereby reducing the levels of hemostatically active factor II, VII, IX and X. Around 99% of warfarin is bound to plasma proteins. It is metabolized mainly by the cytochrome P450 CYP2C9 in the liver and to a lesser extent by other cytochromes including CYP3A4 [71]. The main aspects of VKAs’ pharmacokinetic and pharmacodynamic alterations in elderly patients have already been discussed above. VKAs have been prescribed for the last few decades in the secondary phase of treatment of acute VTE after the initial phase with a parenteral anticoagulant, and also in preventing thromboembolic complications of AF. As a general rule, target INR in standard situations is 2.5 (2.0-3.0).

When introducing VKAs in elderly patients, their higher sensitivity to these medications should be taken into account, and lower initial doses should be prescribed as also recommended in the latest ACCP guidelines [71]. Siguret *et al*. developed and prospectively validated a specific low-dose regimen for warfarin introduction (initial dose of 4 mg/day) in medical elderly inpatients. INR performed after the third dose could reliably predict maintenance dose. This algorithm thus represents a useful practical tool for initiation of warfarin in the elderly [72]. As for other anticoagulants, the greatest fear of physicians is of hemorrhagic complications. This is particularly true for the use of warfarin in AF patients as it is frequently prescribed on a “long term” basis. Variable rates of major hemorrhage have been reported on VKA in clinical routine practice, especially in the elderly [41]. An increased risk of major and particularly intracranial hemorrhage was observed by Fang *et al.* in patients ≥80 years of age with AF whether they were on warfarin or not, suggesting that anticoagulation with warfarin with careful monitoring could be safely used in elderly patients [73]. Only a slight tendency toward an increase in bleeding with age on warfarin prescribed for various indications was shown by Palareti *et al.* with an incidence of 9.9% *versus* 6.6% (p = 0.7) for patients ≥75 years compared to those <70 years. In this latter study however, the rates of intracranial hemorrhage were significantly higher in older than younger anticoagulated patients (1.1% *versus* 0.2%, p = 0.05) [74]. One interesting finding is the higher rate of major hemorrhage on warfarin in the first few months of treatment in patients ≥80 years compared with those <80 years, with an incidence of 13.1 *versus* 4.7 per 100 patient-years, the highest rates being observed during the first three months of treatment [75]. The overall higher rates of bleeding in this latter study could possibly be explained by the fact that it analysed “incident” anticoagulated patients, whereas previous studies included “prevalent” anticoagulated patients potentially representing a selected population of warfarin-tolerant individuals.

To refine the assessment of hemorrhagic risk during the first three months of anticoagulation, a simple bleeding score was developed by Ruiz-Gimenez *et al.* in patients with VTE from the RIETE registry including six clinical or biological items (Table 2) [42]. It identified 20% of patients at very low risk of bleeding (0.1-0.3% at 3 months), and another 5% at high risk (>7% at 3 months). The limitations of this score are its lack of prospective validation and its limited scope to the initial three months, which renders it inapplicable for later “re-assessments” of the benefit-risk ratio of continuing anticoagulation.

For the evaluation of bleeding risk on warfarin in AF patients, the HEMORR2HAGES score was developed by Gage *et al.* [43]. The items included in this score and the annual incidence of major bleeding are presented in Table 2. Another bleeding risk model was developed by Shireman *et al.* for elderly warfarin recipients (>65 years) including eight items: age ≥70 years, gender, remote bleeding, recent bleeding, alcohol/drug abuse, diabetes, anemia, antiplatelet use. The risk of major bleeding was 0.9%, 2.0% and 5.4% for groups with low, moderate and high risk. An interesting fact is that more than 40% of patients in this study were ≥80 years of age, representing a truly geriatric population [76]. Yet another bleeding score was published this year by Pisters et al under the acronym HAS-BLED including the following items: Hypertension (uncontrolled, >160mmHg systolic), Abnormal renal/liver function, Stroke, Bleeding history or predisposition (anemia), Labile INR (therapeutic time in range <60%), Elderly (>65 years), Drugs/alcohol concomitantly (antiplatelets, NSAIDs) [77]. The predictive power of HEMOR2RHAGES and HAS-BLED scores was compared in patients from the Euro Heart Survey in this same paper. For patients on oral anticoagulation alone, these scores showed similar discriminatory properties with c-statistics of 0.64 and 0.69 respectively [77]. 

Intensity of anticoagulation with VKAs is one of the major determinants of bleeding, an INR >3.0 doubling the risk compared to an INR within the therapeutic range of 2.0-3.0 [41]. Furthermore, hemorrhagic risk increases exponentially with INR values >4.5 regardless of patient’s age [74]. Interestingly, the risk of intracranial hemorrhage does not seem different between patients with an INR <2.0 and patients with an INR of 2.0-3.0 [74,78]. This suggests that a well controlled anticoagulation can offer an acceptable safety profile even in elderly patients. Therefore, when a patient is evaluated for benefit-risk ratio of continuing VKAs, the quality, regularity, and ease of management of anticoagulation should be taken into account. 

As for any other medication prescribed on a long term basis, patient’s adherence to treatment is a major issue for long term oral anticoagulation in all age groups. Kimmel *et al*. prospectively observed 136 patients (aged 48.5 to 70 years old) from three anticoagulation clinics by using electronic medication bottle caps that could record each opening of the warfarin bottle over a mean period of 32 weeks. They showed that 92% of patients had at least one missed or extra bottle opening, and 36% omitted more than 20% of their bottle openings. The authors also demonstrated a significant influence of poor adherence on anticoagulation control [79]. In elderly patients, in addition to the numerous pharmacokinetic and pharmacodynamic interactions mentioned above, compliance probably also has a major impact on the quality of anticoagulation, frequently assessed by the percentage of INR values in the therapeutic interval and expressed as the “time in the therapeutic range” (TTR). Even in clinical trials, the TTR is often no more than 60-65% (as an example, the TTR in the warfarin arm of the RE-LY study discussed below was 64%) [80]. In cohort studies of elderly patients reflecting “real life” patients, the TTR is even much lower. Kagansky *et al.*, for instance, showed a TTR of 35.4% in patients ≥80 years followed up for a mean period of 28.8 (±36.3) months after discharge from hospital with an indication for oral anticoagulation. Interestingly, the TTR was significantly higher in patients who had received satisfactory explanation on oral anticoagulation (TTR 45.1%) and very low in those who did not receive any explanation (TTR 20%), emphasising the importance of patient education, even in the very old [44]. 

Deciding whether he will do more good or harm to his patient by prescribing or not prescribing anticoagulation is often a real puzzle for the physician taking care of elderly patients. The tendency to overestimate bleeding risk in geriatric patients who would be candidates for anticoagulation for AF could be of “emotional” origin, physicians feeling personally responsible for a hemorrhagic complication of treatment, whereas thromboembolic complications are considered as fate [81]. In reality, the difficulty in elderly patients lies in the fact that those at highest risk for bleeding are those who would have the greatest benefit from anticoagulation. Thromboembolic and hemorrhagic prediction scores can be useful tools in helping physicians balance the benefit-risk ratio of anticoagulation in individual patients. Besides, patient’s preferences should also always be taken into account.

## 5. Newer Anticoagulants

**Figure 1:**
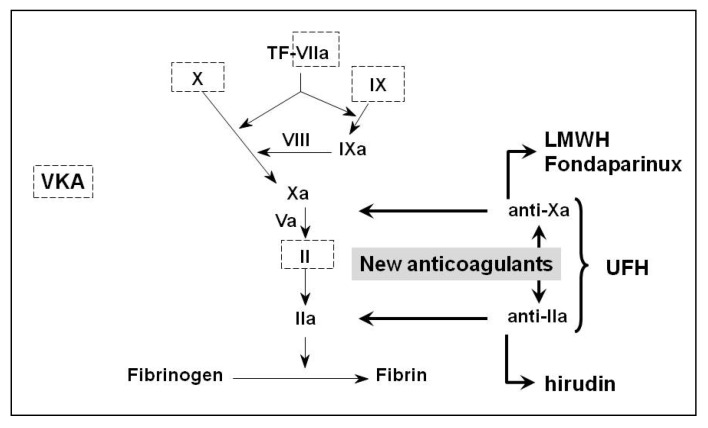
Sites of action of anticoagulants in the coagulation cascade.

In view of the limitations of currently available anticoagulants, it is easy to understand why newer anticoagulants are eagerly awaited. In particular, a new oral anticoagulant for long term treatment could change millions of patients’ quality of life if it proves to be at least as effective as VKAs without most of their disadvantages. VKAs have indeed been the only oral anticoagulants available for the last 65 years, but research in the domain of novel anticoagulant has been particularly active over the last few years. The specificity of newer anticoagulants is the fact that they target selectively one step in the coagulation cascade as opposed to VKAs (Figure 1). In this section, the two main classes of newer anticoagulants are presented. It should be noted that the recent studies on newer oral anticoagulants are not specifically focused on the elderly and the proportion of elderly patients is reduced by the exclusion criterion of calculated creatinine clearance <30 mL/min, so that the results of these trials should be applied with caution to geriatric patients. Some specific concerns that could arise with these new agents in elderly patients are discussed hereafter. 

### 5.1. Direct Thrombin (Factor IIa) Inhibitors

The choice of thrombin as a target for new anticoagulants is logical. Indeed, thrombin is the final effector in the coagulation cascade converting fibrinogen to fibrin. It also amplifies its own generation and interferes in the coordination of platelet activation and aggregation with coagulation. Current examples of anti-factor IIa molecules are hirudin and argatroban, parenteral drugs approved for the treatment of heparin-induced thrombocytopenia. Proof of concept that an oral thrombin inhibitor with no need for monitoring could be as effective and safe in terms of major bleeding than standard therapy was set by trials on ximelagatran. In 2005, the THRIVE investigators demonstrated the non-inferiority of ximelagatran compared to enoxaparin followed by warfarin for proximal DVT [82]. This drug also proved equivalent to standard anticoagulation in several other settings including VTE prophylaxis after orthopedic surgery and stroke prevention in AF [83]. Unfortunately, ximelagatran was withdrawn from the world market after temporary licensing in Europe, because of concerns about its potential hepatic toxicity.

Currently, dabigatran etexilate is the new oral anticoagulant which is in the most advanced stages of development, with recent publication of the results of phase III clinical trials in AF and VTE. It is a prodrug administered orally twice daily. Because of its high lipophilic nature, it is formulated with tartaric acid in order to increase gastro-intestinal absorption. Oral bioavailability is however extremely low (6%). It is rapidly converted to dabigatran and plasma peak levels are achieved 2 hours after ingestion. Importantly, dabigatran etexilate metabolism to its active form does not involve P450 cytochromes, especially CYP3A4 and CYP2C9, reducing the potential for drug-drug interactions. Only 25-35% is bound to albumin in plasma. The drug is mainly cleared via the kidneys (80%) with a half-life of 14-17 h (Table 4). Its pharmacokinetics is linear, dose-dependent and highly predictable [84]. As the transporter P-glycoprotein (P-gp) in the intestinal epithelium interferes with dabigatran etexilate absorption, potent inhibitors of P-gp significantly interact with dabigatran by increasing its concentration [85]. Of these, quinidine has been shown to double bioavailability of dabigatran and its concomitant use with dabigatran is therefore contra-indicated by the manufacturer. Other currently prescribed drugs such as amiodarone, verapamil and clarithromycin are also inhibitors of P-gp. The manufacturer suggests dabigatran dose reduction when amiodarone is prescribed concurrently in AF patients. Of note, dabigatran use has not been associated with hepatotoxicty so far. One drawback is the absence of antidote, and in case of melagatran, recombinant factor VIIa had shown a limited ability to reverse the anticoagulant effect [86].

**Table 4:** Characteristics of new oral anticoagulants.

Characteristics	Dabigatran etexilate	Rivaroxaban	Apixaban
Target	Thrombin (factor IIa)	Factor Xa	Factor Xa
Dosing pattern (therapeutic anticoagulation)	Fixed, b.i.d.	Fixed, o.d.	Fixed, b.i.d.
Monitoring necessary	No	No	No
Prodrug	Yes	No	No
Bioavailability (%)	6	>80	50
Half-life (hours)	14–17	5–13	8-15
Clearance	80% renal 20% biliary	1/3 renal: unchanged 1/3 renal: inactive 1/3 biliary	25% renal

Phase III trials of dabigatran etexilate in major orthopedic surgery were published between 2007 and 2009. In summary, non-inferiority of both doses of dabigatran etexilate (150 mg o.d. and 220 mg o.d.) were demonstrated compared to enoxaparin 40 mg o.d. (European regimen) [87,88], but not compared to enoxaparin 30 mg b.i.d. (North American regimen) [89]. Major bleeding episodes were rare in all these studies, with no significant difference between study arms. However, subgroup analysis of pooled data from these trials revealed a higher bleeding risk in patients with moderate renal impairment (CrCl ≥ 30 mL/min to <50 mL/min) and in patients older than 75 years [90], for whom the daily dose of 150 mg is therefore recommended. For anticoagulation in the therapeutic range, dabigatran etexilate was compared to warfarin in the RE-LY study including AF patients and in the RE-COVER study including VTE patients. In terms of efficacy against thromboembolism and safety considering rates of major hemorrhage, RE-LY showed that dabigatran etexilate at 110 mg b.i.d. was non-inferior to warfarin in terms of efficacy and safer, and dabigatran at 150 mg b.i.d. was superior to warfarin in efficacy, but associated with similar rates of major hemorrhage [80]. RE-COVER compared dabigatran etexilate 150 mg b.i.d. with warfarin in acute VTE after an initial week of treatment with a parenteral drug. Non-inferiority criteria were met for dabigatran compared to warfarin regarding the primary outcome defined as symptomatic and objectively confirmed VTE or death related to VTE (2.4% *versus* 2.1%; *p*-value for non-inferiority <0.001) [91]. 

Some concerns could probably arise with this medication at therapeutic doses in elderly patients. First, dabigatran etexilate is associated with a significantly higher rate of dyspepsia than warfarin, and this effect seems more prominent in older patients. Indeed, mean age of patients varied widely between RE-LY and RE-COVER studies as it is usual between AF and VTE trials (mean age 71.5 years in RE-LY *versus* 55 years in RE-COVER). While the absolute incidence of dyspepsia is quite low in all groups in RE-COVER (3.1% for dabigatran *versus* 0.7% for warfarin), this seems much more of an issue in older patients of the RE-LY study with an incidence of dyspepsia of more than 11% in patients on dabigatran etexilate *versus* 5.8% on warfarin. The overall higher incidence of dyspepsia in RE-LY compared to RE-COVER could also be related to the longer observation period (2 years *versus* 6 months). This side effect could possibly interfere with elderly patients’ long term compliance. The other concern in geriatric patients is obviously the renal route of elimination. Patients with CrCl < 30 mL/min were excluded from the RE-COVER and RE-LY studies. However, accumulation of dabigatran has already been observed in mild and moderate renal impairment with a ratio of accumulation of 1.4 and 1.8 respectively compared to normal renal function [92]. Finally, the twice daily regimen represents a negative point for its use in elderly patients who already tend to have more tablets to take each day than younger patients.

### 5.2. Direct Factor Xa Inhibitors

Factor Xa is also an interesting target for new oral anticoagulants. It indeed assembles with factor Va on the surface of activated platelets, forming the prothrombinase complex which is a potent activator of prothrombin. Factor Xa inhibitors reversibly block the active site of factor Xa, without binding to antithrombin, hence their naming as *direct* inhibitors. After oral administration, their bioavailability is high (>80%), and peak concentration is reached in 2-4 h after ingestion. Up to 92-95% is bound to albumin. Elimination is through many pathways: 1/3 is metabolized via CYP3A4/3A5 and CYP2J2 in the liver and excreted in the feces, 1/3 is excreted in the urine in the active form and 1/3 in a partly metabolized form. Half-lives of different anti-Xa inhibitors vary, with a range of 7-15 h [85] (Table 4). 

Currently rivaroxaban and apixaban are the two direct oral factor Xa inhibitors undergoing phase III clinical trials. In VTE prophylaxis after major orthopedic surgery studies (RECORD studies), with a non-inferiority design of rivaroxaban 10 mg o.d. *versus* enoxaparin 40 mg o.d. or 30 mg b.i.d., rivaroxaban actually showed superiority in terms of reduction of the composite endpoint of total VTE (symptomatic and asymptomatic DVT and non-fatal PE) and all-cause mortality, without increasing the risk of major bleeding [93,94,95]. Results of the phase III trial in DVT have recently been presented at the European Society of Cardiology, and confirmed non-inferiority of rivaroxaban *versus* standard therapy in terms of protection against thromboembolism, without any difference in the risk of major bleeding or clinically relevant non-major bleeding. The ROCKET study of rivaroxaban *versus* warfarin in AF, recently presented at the American Heart Association congress, showed non-inferiority of rivaroxaban compared to warfarin by intention to treat (rates of stroke or non-CNS systemic embolism 2.12% *versus* 2.42%; p <0.001 for non-inferiority), with similar rates of major and non-major clinically relevant bleeding events, but fewer intracranial hemorrhages. Of note, the time in therapeutic range in the warfarin group was 55% in this study.

Apixaban has similar characteristics to rivaroxaban, in terms of half-life, percentage of renal elimination (1/4), potential drug-drug interactions because of metabolism by CYP3A4, but a lower bioavailability (52%). In VTE prophylaxis after knee arthroplasty, although thromboemoblic event rates were similar between apixaban 2.5 mg o.d. and enoxaparin 30 mg b.i.d. (ADAVANCE 1), the preset non-inferiority criteria were not met because of a lower than expected event rate. Apixaban was however associated with a lower rate of bleeding complications. When compared to enoxaparin 40 mg o.d. (ADVANCE 2), apixaban was more effective with a non significant trend towards less bleeding. The results of AVERROES trial comparing apixaban 2.5 mg b.i.d. to aspirin 81-324 mg o.d. in AF were recently presented at the European Society of Cardiology 2010. This study was terminated prematurely due to superior efficacy of apixaban without any increase in major or intracralnial bleeding. Other phase III trials of apixaban in AF and VTE are currently ongoing. Phase III trials on still another oral factor Xa inhibitor, edoxaban, have been completed for prevention of VTE in major orthopedic surgery and hip fracture, and trials on VTE treatment and AF are ongoing. Of note, there has been no concern about hepatotoxicity with oral anti-Xa inhibitors so far. The rates of potential (non hemorrhagic) adverse effects that may be particularly relevant in elderly patients will need to be thoroughly analyzed when results from these different studies will be published. Furthermore, one concern that could arise with this category of drugs in elderly patients is the potential for drug-drug interactions in patients with multiple other medications.

Overall, there is no doubt that new anticoagulants offer major advantages over warfarin and are much closer to the image of the ideal anticoagulant. In elderly patients, the possibility of taking a medication that does not need monitoring and is not subject to fluctuation with nutritional intake or to interaction with other medications is priceless. Each of the two categories of newer anticoagulants has its own advantages and drawbacks if specificities of the geriatric population are considered. If the positive results demonstrated in phase III trials are confirmed in long term use in clinical practice, these drugs will certainly become the oral anticoagulants of choice in the near future.

## 6. Conclusions

Elderly patients are both at high thromboembolic and hemorrhagic risk. In order to offer these patients effective protection against thromboembolism with maximum safety, many specific considerations need to be taken into account. The overall tendency among physicians is an underuse of anticoagulants in elderly patients in almost all indications because of an overestimation of hemorrhagic risk. Clinical scores can partly help physicians to make an objective evaluation of thromboembolic and hemorrhagic risks. Nevertheless, the most difficult task remains assessment of the benefit-risk *balance* of anticoagulation in an individual patient, as those at highest hemorrhagic risk are often those that would have the greatest benefit from anticoagulation. All currently available anticoagulant agents can be used in elderly patients provided some precautions are observed. If the positive results of newer anticoagulants’ trials continue to be confirmed in the general and elderly population of patients, these could probably supplant most of the currently used anticoagulant modalities.
